# Immunological Microenvironment Alterations in Follicles of Patients With Autoimmune Thyroiditis

**DOI:** 10.3389/fimmu.2021.770852

**Published:** 2021-11-17

**Authors:** Ning Huang, Dandan Liu, Ying Lian, Hongbin Chi, Jie Qiao

**Affiliations:** ^1^ Center for Reproductive Medicine, Department of Obstetrics and Gynecology, Peking University Third Hospital, Beijing, China; ^2^ National Clinical Research Center for Obstetrics and Gynecology, Peking University Third Hospital, Beijing, China; ^3^ Key Laboratory of Assisted Reproduction (Peking University), Ministry of Education, Beijing, China; ^4^ Beijing Key Laboratory of Reproductive Endocrinology and Assisted Reproductive Technology, Peking University Third Hospital, Beijing, China

**Keywords:** autoimmune thyroiditis, ovarian microenvironment, follicle development, IFNγ-CXCL9/10/11-CXCR3^+^ T lymphocyte, follicular fluid

## Abstract

Autoimmune thyroiditis (AIT) is the most prevalent autoimmune endocrine disease, with a higher incidence in women than in men. Immunological abnormalities may lead to the impairment of ovarian folliculogenesis; however, whether the presence of AIT affects immunological microenvironment in follicles remains controversial. We performed a cross-sectional study including 122 patients, aged 20–40 years, who underwent IVF/ICSI treatment owing to isolated male or tube factor infertility. Patients were divided into AIT and control groups according to clinical presentation, thyroid function, and thyroid autoantibody measurements. Follicular fluid was collected and the distribution of cytokines/chemokines in follicular fluid was measured by flow cytometry using multiplex bead assays between the two groups. Based on differences in levels of intrafollicular chemokines and cytokines between the AIT and control groups, the relevant inflammatory cascade was further demonstrated. Among the 12 chemokines analyzed, three (CXCL9, CXCL10, and CXCL11) showed significantly elevated levels in the follicular fluid of patients with AIT. Among the 11 cytokines detected, compared with those in the control group, significantly higher levels of IFNγ were observed in patients with AIT. IFNγ dose-dependently stimulated the expression and secretion of CXCL9/10/11 in cultured primary granulosa cells. The percentage of CXCR3^+^ T lymphocytes was significantly elevated in the follicular microenvironment of patients with AIT. We concluded that the IFNγ-CXCL9/10/11-CXCR3^+^ T lymphocyte inflammatory cascade is activated in the follicular microenvironment of patients with AIT. These findings indicate that a considerable immune imbalance occurred in the follicular microenvironment of patients with AIT.

## Introduction

Autoimmune thyroiditis (AIT) is an organ-specific autoimmune disorder characterized by the production of autoimmune thyroid antibodies and the infiltration of self-reactive lymphocytes into the thyroid, resulting in the destruction of thyrocytes and hypothyroidism. T cell-mediated immune disorders play an important role in the pathogenesis of AIT. In patients with AIT, both in the peripheral blood and in the thyroid gland, the balance between Th1 and Th2 cells is skewed toward Th1 cells and cytotoxic T lymphocytes are considerably activated, which triggers a dramatic activation of the immune response. Meanwhile, pro-inflammatory cytokines such as interferon-gamma (IFNγ) and IL-17 were significantly elevated. Pro-inflammatory chemokines CXCL9, CXCL10, and CXCL11 were also activated in patients with AIT, which further promoted the migration of pro-inflammatory Th1 lymphocytes into the thyroid and aggravated the destruction of the thyroid gland (1–4).

Immunological abnormalities may lead to the impairment of ovarian folliculogenesis; however, whether the presence of AIT affects follicular development and ovulation remains controversial and the relevant studies are limited. Our previous large-scale retrospective cohort study found that after adjusting for thyroid function and ovarian reserve markers, the number of oocytes retrieved during *in vitro* fertilization/intracytoplasmic sperm injection (IVF/ICSI) treatment significantly decreased in infertile women with positive thyroid antibodies. This suggests a possible role of AIT in follicular development; however, the mechanism remains unclear (5).

Follicle development and oocyte maturation depend heavily on the follicle fluid, which is derived from blood infiltration and ovarian secretion. An altered follicular microenvironment may directly damage follicle recruitment and growth. A large number of cytokines/chemokines are activated in follicular fluid and interact with each other to form a network that regulates folliculogenesis, oocyte maturation, and luteinization (6). Recent studies have reported an association between abnormal elevation of specific cytokines/chemokines and follicle developmental disorders (7, 8).

This study aimed to investigate the distribution of intrafollicular cytokines/chemokines and the activation of relevant inflammatory pathways in patients with AIT compared to those without AIT.

## Materials and Methods

### Ethical Approval

The study was approved by the Peking University Third Hospital Medical Science Research Ethics Committee.

### Patients

The study consisted of 122 patients, aged 20–40 years, who underwent IVF/ICSI treatment owing to isolated male or tube factor infertility. Patients were divided into AIT and control groups according to clinical presentation, thyroid function, and thyroid autoantibody measurements. The reference values for serum thyroid-stimulating hormone (TSH) were 0.55–4.78 uIU/mL, and those for serum-free thyroxin (FT4) were 0.89–1.80 ng/dL. The detection range for thyroglobulin antibody (TGAb) was 15–500 IU/mL, and that for thyroid peroxidase antibody (TPOAb) was 28–1300 IU/mL. A value >60 IU/mL was defined as positive for TGAb or TPOAb. Patients positive for TGAb and/or TPOAb were placed in the AIT group. The levels of TSH and FT4 in all patients were required to be within the normal reference range prior to controlled ovarian stimulation; thus, 14 patients that were thyroid antibody-positive received oral levothyroxine owing to abnormal TSH and FT4 levels. Patients with a history of other reproductive diseases, such as polycystic ovarian syndrome or endometriosis; a history of other thyroid diseases, such as hyperthyroidism or thyroid cancer; abnormal results on parental karyotyping; other endocrinological diseases, such as hyperprolactinemia or diabetes; or positive tests for antinuclear antibodies or lupus anticoagulants were excluded.

### IVF/ICSI Procedure and Follicle Fluid Collection

All patients underwent standardized protocols for controlled ovarian stimulation (COS) and oocyte retrieval. The individualized dose of recombinant follicle-stimulating hormones for inducing COS was decided based on the patient’s age, body mass index (BMI), antral follicle count, and anti-Mullerian hormone levels. Two hundred and fifty micrograms of recombinant human chorionic gonadotropin (HCG; Eiser, Serono, Germany) was administered to trigger oocyte maturation when at least two follicles reached 18 mm. Oocyte retrieval was performed 34–36 h after HCG administration. Follicular fluid without visible blood contamination was aspirated from the first and largest follicle, collected separately and centrifuged at 800 g for 10 min. Supernatants were collected and stored at −80°C until further analysis.

### Cytokine/Chemokine Detection Assay

Concentrations of cytokines/chemokines in the follicle fluid were measured using flow cytometry with multiplex bead assays, and the procedures were performed using the Legendplex Multi-Analyte Flow Assay Kit according to the manufacturer’s instructions (BioLegend). Cytokine plexes include IL-2, IL-4, IL-6, IL-9, IL-10, IL13, IL-17A, IL-17F, IL-22, IFNγ, and TNFα. The chemokine plexes included CCL2, CCL3, CCL4, CCL5, CCL11, CCL17, CXCL1, CXCL5, CXCL8, CXCL9, CXCL10, and CXCL11.

### Chemokine Secretion Assay

Primary granulosa cells were collected from follicular aspirates and purified *via* centrifugation at 600 g for 30 min using the Ficoll gradient method. For chemokine secretion assays, primary granulosa cells were seeded onto six-well plates in growth medium (Dulbecco’s modified Eagle’s medium/Ham’s F-12 nutrient mixture containing 10% fetal bovine serum). The growth medium containing leukocytes was removed after 24 h, granulosa cells were washed in PBS, and incubated in serum-free growth medium. Cells were incubated for 24 h with different doses of IFNγ (R&D Systems; 500, 1000, 5000, and 10,000 IU/mL). The supernatant was removed after 24 h and stored at −20°C until required for the chemokine assay.

### Real-Time Polymerase Chain Reaction (RT-PCR)

Total RNA was extracted from cultured primary granulosa cells using TRIzol reagent (Life Technologies) according to the manufacturer’s instructions. RNA was reverse transcribed into first-strand cDNA using a cDNA synthesis kit (Thermo Fisher Scientific). Reverse transcription quantitative RT-PCR was performed on an Applied Biosystems Quant Studio3 Real-Time PCR System in 96 well optical reaction plates. The following primers were used: β-actin F, TGCCCATCTACGAGGGGTAT; β-actin R, CTTAATGTCACGCACGATTTCC; CXCL9 F, CATCATCTTGCTGGTTCTGATTG; CXCL9 R, GATTGTAGGTGGATAGTCCCTTG; CXCL10 F, GCTGCCTTATCTTTCTGACTCT; CXCL10 R, GACCTTGGATTAACAGGTTGATTAC; CXCL11 F, ATGAGTGTGAAGGGCATGG; CXCL11 R, TATGCAAAGACAGCGTCCTC.

### Flow Cytometry

The percentage of intrafollicular CXCR3^+^ T lymphocytes was detected in 14 patients with AIT and in the 12 control patients. The remaining follicle fluid in each patient was collected after the oocytes were removed and centrifugated at 800 g for 10 min. Cells were aspirated and washed with Dulbecco’s PBS. The cells were treated with lysis buffer (BD Biosciences) to remove red blood cells. Approximately 2 × 10^6^ cells were filtered through a 70-μm mesh to remove clumps of cells. Cells were incubated with 100 μL PBS containing CD45 microbeads and CD45 antibody. CD45^+^ cells were isolated using a MACS separator (Miltenyi Biotec). Cells were further phenotyped by staining with CD3, CD4, and CD183 (CXCR3) and counted using a flow cytometer (BD FACSCelesta™).

### Statistical Analysis

Statistical analysis was performed using SPSS version 24.0 software (SPSS Inc, Chicago, IL, USA) and GraphPad Prism 6.0 (La Jolla, CA, USA). Comparisons of continuous data among groups were performed using ANOVA or non-parametric tests. A two-tailed P value of <0.05 was defined as statistically significant.

## Results

The baseline characteristics of patients with AIT and the control group are shown in **Table 1**. The distributions of age and BMI were similar between the two groups. No significant difference was observed in FT4 and TSH levels in serum or follicular fluid of patients with or without AIT. Significantly higher levels of TGAb and TPOAb in serum and follicular fluid were detected in patients with AIT than in the control group (P <0.001). Thyroid antibodies in serum and follicular fluid were absent in the control group.

**Table 1:** Baseline characteristics in patients with and without autoimmune thyroiditis (AIT).

Characteristics	AIT group (n = 66)	Control group (n = 56)	P value
Age	33.1 (3.9)	32.1 (4.2)	0.15
Body mass index	22.3 (3.5)	22.0 (3.1)	0.64
Serum FT4	1.2 (0.1)	1.2 (0.1)	0.19
Serum TSH	2.4 (1.0)	2.3 (0.9)	0.60
Serum TGAb	172.0 (77.1–294.3)	15.0 (15.0–18.8)	<0.001
Serum TGAb positivity (%)	80.3	0	<0.001
Serum TPOAb	289.3 (33.0–1300.0)	28.0 (28.0–30.2)	<0.001
Serum TPOAb positivity (%)	68.2	0	<0.001
FF FT4	1.2 (0.1)	1.2 (0.1)	0.61
FF TSH	2.6 (1.1)	2.5 (1.1)	0.83
FF TGAb	118.2 (44.5–210.2)	15.0 (15.0–15.8)	<0.001
FF TGAb positivity (%)	69.7	0	<0.001
FF TPOAb	198.7 (41.9–1300.0)	36.5 (31.6–42.1)	<0.001
FF TPOAb positivity (%)	71.2	0	<0.001

FT4, free thyroxin; TGAb, thyroglobulin antibody; TPOAb, thyroid peroxidase antibody; TSH, thyroid stimulating hormone; FF, follicular fluid.

Continuous variables were expressed as medians (interquartile range) when the data did not follow a Gaussian distribution, and as means (standard deviation) for normally distributed data.

**Figure 1:**
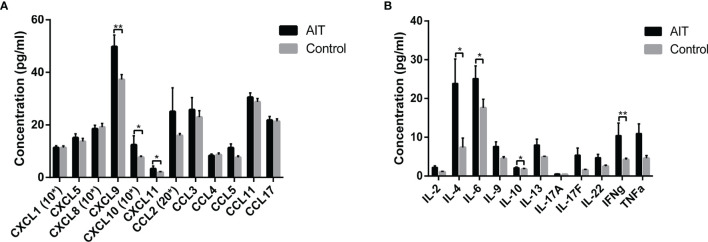
Distribution of cytokines and chemokines in follicle fluid obtained from patients with and without AIT. Among the 12 chemokines analyzed, concentrations of CXCL9, CXCL10, and CXCL11 were significantly higher in patients with AIT than those in the control group **(A)**. Bars represent the mean ± SEM. *P < 0.05 and **P < 0.01 by the Mann-Whitney U test. Among the 11 cytokines analyzed, IL4, IL-6, IL-10, and IFNγ concentrations were significantly higher in patients with AIT than in the control group **(B)**. Bars represent the mean ± SEM. *P < 0.05 and **P < 0.01 by the Mann-Whitney U test. *10, data needed to be multiplied by 10; *20, data needed to be multiplied by 20.

We analyzed the distribution of chemokines and cytokines in the follicular fluid between the AIT and control groups (**Figure 1**). Among the 12 chemokines analyzed, only the concentrations of three, CXCL9, CXCL10, and CXCL11, were significantly elevated in the follicular fluid of patients with AIT. There was no significant difference in the levels of other chemokines between the two groups. Among the 11 cytokines analyzed, significantly higher levels of IFNγ were observed in patients with AIT than in the control group (P <0.01). Marginal yet significantly higher levels of IL4, IL-6, and IL-10 were also observed in patients with AIT than in the control group (P <0.05). There was no significant difference in the levels of other cytokines between the two groups.

**Figure 2:**
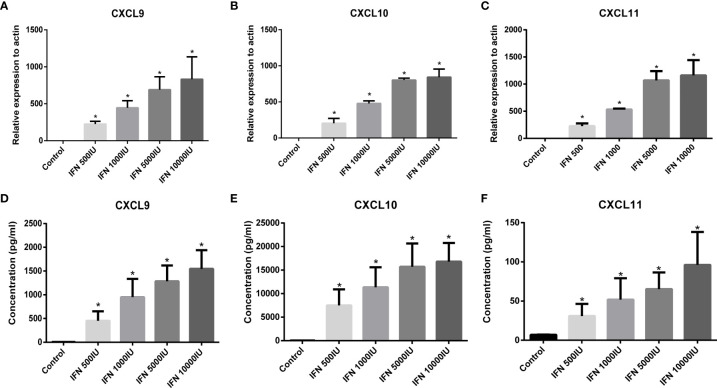
Expression of CXCL9 **(A)**, CXCL10 **(B)**, and CXCL11 **(C)** in primary granulosa cells stimulated by IFNγ (IFN-g) was detected by RT-PCR. Expression of CXCL9/10/11 in granulosa cells was absent under basal conditions (control) and was significantly stimulated by increasing doses of IFNγ (ANOVA, P < 0.05). Bars represent the mean ± SEM. *P < 0.05 vs control by the Bonferroni-Dunn test. Concentration of CXCL9 **(D)**, CXCL10 **(E)**, and CXCL11 **(F)** in the culture supernatant from granulosa cells stimulated by IFNγ. The release of CXCL9/10/11 from granulosa cells was absent under basal conditions (control) and was significantly stimulated by increasing doses of IFNγ (ANOVA, P <0.05). Bars represent the mean ± SEM. *P < 0.05 vs control by the Bonferroni-Dunn test.

IFNγ has been evidenced as an effective inducer for the activation of CXCL9/10/11 in thyrocytes, however, the role of IFNγ in intrafollicular granulosa cells remains unclear. Based on differences in distribution of intrafollicular chemokines and cytokines between the AIT and control groups, elevated CXCL9/10/11 secretion may be induced by increased IFNγ in the follicular microenvironment of patients with AIT. To investigate whether intrafollicular IFNγ stimulates the secretion of CXCL9/10/11 in granulosa cells, primary granulosa cells were cultured *in vitro* and treated with different doses of IFNγ. As shown in **Figure 2**, IFNγ dose-dependently induced the expression and secretion of CXCL9/10/11 in primary granulosa cells.

**Figure 3:**
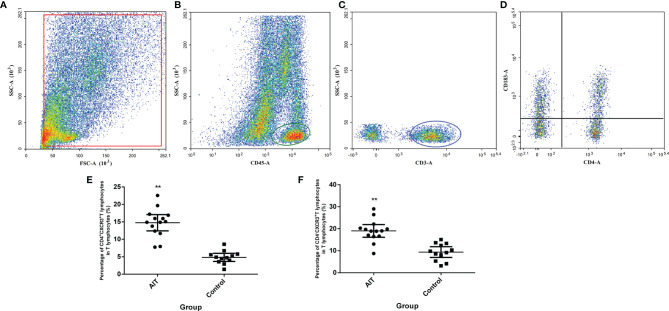
Detection of CXCR3^+^ T lymphocytes in the follicular microenvironment. Cells were enriched from follicular fluid, and CD45^+^ cells were positively selected by MACS cell separation. Separated cells were directly incubated with conjugated anti-CD3, CD4, and CXCR3 antibodies and counted by flow cytometry **(A)**. CD45^+^ cells were gated **(B)** and CD3^+^ cells were gated from CD45^+^ cells **(C)**, the expressions of CXCR3 in CD4^+^ and CD4^-^T cells were gated from CD3^+^ cells **(D)**. **(E, F)** The percentage of CD4^+^CXCR3^+^ and CD4^-^ CXCR3^+^ T lymphocytes. **P < 0.01 by the Mann-Whitney U test.

CXCL9/10/11 shares the common receptor CXCR3 (CD183), which is primarily expressed in T lymphocytes (9, 10). To investigate whether elevated intrafollicular CXCL9/10/11 in patients with AIT promoted the infiltration of T lymphocytes into follicle fluid, we measured the percentage of T lymphocytes expressing CXCR3 in follicular fluid from 14 patients with AIT and compared it with 12 control patients using flow cytometry. As shown in **Figure 3**, of the CD45^+^CD3^+^ T lymphocytes, the proportions of CD4^+^CXCR3^+^ and CD4^−^CXCR3^+^ T lymphocytes in the AIT group were significantly higher than those in the control group.

## Discussion

**Figure 4:**
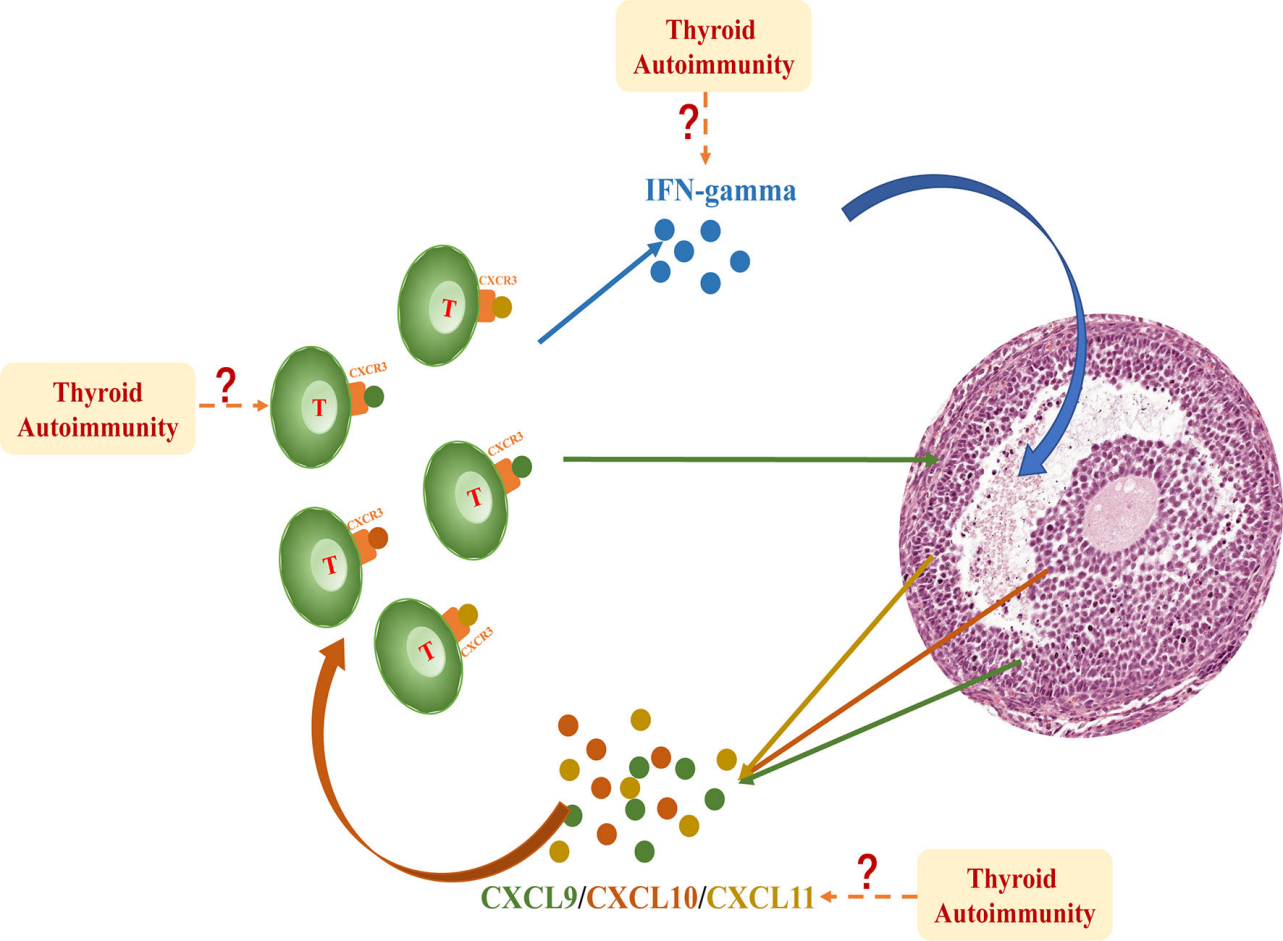
IFNγproduced by T lymphocytes dose-dependently induced the expression and secretion of CXCL9/10/11 in primary granulosa cells. These chemokines bind to their receptor CXCR3 and further promote the migration of T lymphocytes into the follicle, thereby perpetuating an inflammatory cascade in the follicular microenvironment. This intrafollicular response is significantly activated in patients with AIT; however, the initiation of this cascade is still unclear. The excess influx of inflammatory cells and factors into the follicle fluid may be involved in this mechanism.

Our study is the first to demonstrate the substantial activation of IFNγ-CXCL9/10/11-CXCR3^+^ T lymphocytes in the ovarian microenvironment of patients with AIT. This cascade included a classical inflammatory response: IFNγ secreted by T lymphocytes stimulated the release of CXCL9/10/11 from granulosa cells. CXCL9, CXCL10, and CXCL11 belong to the chemokine family and share the common receptor CXCR3, which is particularly expressed in T lymphocytes. Elevation of CXCL9/10/11 in follicle fluid attracts CXCR3^+^ T lymphocytes to infiltrate the follicle. T lymphocytes that remained in the follicle further increased the secretion of IFNγ, which constitutes an intact response loop in the follicular microenvironment (**Figure 4**).

There is increasing evidence to demonstrate the role of intrafollicular inflammation in the pathogenesis of reproductive diseases. A previous study analyzed the association between follicular pro-inflammatory cytokines/chemokines and infertility related diseases and showed that different types of cytokines/chemokines were activated in patients with different reproductive diseases. For example, higher follicular MIP-1α (CCL3) and CD44 were correlated with polycystic ovary syndrome and IL-23, IFN-γ, and TNF-α correlated with endometriosis (11). An imbalance between pro-inflammatory cytokines such as IFNγ and TNFα and anti-inflammatory cytokines such as IL-10 has also been reported to play a critical role in the pathological mechanism of premature ovarian insufficiency (8). Our study showed a significant difference in the distribution of intrafollicular chemokines and cytokines between the AIT and control groups, suggesting the presence of an activated abnormal immune response in the follicular microenvironment in patients with AIT.

Whether increased levels of intrafollicular CXCL9/10/11 affect follicle development is still unclear; however, CXCL9/10/11 has been reported to inhibit angiogenesis. Several studies have reported that increased levels of CXCL9/10/11 significantly inhibit blood vessel formation and endothelial cell motility (12, 13). In the ovary, angiogenesis is essential for folliculogenesis and ovulation. A rich vascular network surrounding the theca cell layer provides sufficient nutrients and oxygen to support follicle growth, and suppression of normal thecal vasculature leads to considerably reduced thecal thickness, decreased granulosa cell proliferation, prevention of ovulation, and increased rate of follicle atresia (14, 15). Elevated levels of intrafollicular CXCL9/10/11 in patients with AIT may indirectly block follicle development by inhibiting ovarian angiogenesis.

CXCL9/10/11 belongs to a family of chemokines with the capacity to attract circulating leukocytes to inflammatory sites by binding to their specific receptor, CXCR3. Prior to ovulation, only a few resident immune cells were present in vessels around the thecal cells. Upon LH surge or stimulation by HCG, the basal lamina is disrupted and several capillaries form and extend into the granulosa cell compartment, with numerous infiltrating leukocytes and a large number of cytokines are secreted into the follicle fluid (16). The association between the infiltration of leukocytes and ovulation is not completely clear, and the complexity of the inflammatory response and leukocyte types makes it difficult to investigate the role that a specific type of leukocyte plays in follicle fluid. A previous study reported that perfused LH and leukocytes in rat ovaries produced a higher number of ovulations; however, perfused leukocytes alone failed to induce ovulation, suggesting that the influx of leukocytes plays a synergetic, yet not necessary, role in ovulation (17). In our study, elevated CXCL9/10/11 levels enhanced the migration of T lymphocytes *via* CXCR3 and led to a higher proportion of intrafollicular T lymphocytes in patients with AIT, which may aggravate the regional inflammatory response.

In our study, the initiation of the IFNγ-CXCL9/10/11-CXCR3^+^ T lymphocyte cascade in the follicular microenvironment remains elusive. We provided two possible hypotheses. First, excess infiltration of circulating CXCL9/10/11 or IFNγ from blood in patients with AIT may affect the cascade. Several studies have demonstrated that CXCL9/10/11 is dramatically increased in the development of AIT. Kimura et al. established a transgenic mouse model with features similar to those of human AIT, which aberrantly expresses IFNγ within the thyroid gland. Compared with wild-type littermates, a transgenic mouse model exclusively expressed CXCL9 and CXCL11, and showed increased expression of CXCL10, which verified that IFNγ stimulated the secretion of CXCL9/10/11 from the thyroid gland (3). A study showed that circulating CXCL9 and CXCL10 levels were significantly elevated in patients with AIT compared with normal controls or patients with multinodular goiter (1). Another study found that circulating CXCL9 and CXCL11 levels were significantly elevated in patients with AIT compared with euthyroid controls or patients with multinodular goiter (18). Circulating IFNγ, the upstream stimulator of CXCL9/10/11, also increased in patients with AIT (4).

Second, local activation of the inflammatory response may be another hypothesis for the initiation of this cascade. A previous study identified thyroid peroxidase using immunocytochemistry in granulosa cells of the human ovarian follicle and provided a hypothesis that the human ovarian follicle may be an independent thyroid-hormone-producing unit (19). Here, thyroid antibodies were detected in the follicular fluid of patients with AIT, with a similar concentration in the serum (**Table 1**). The presence of thyroid peroxidase and thyroid antibodies in follicular fluid may provide a hypothesis for activation of the intrafollicular antibody-mediated immune response.

Although our findings demonstrate an immunological alteration occurred in follicles of patients with AIT, whether the substantial activation of inflammatory cascade affect folliculogenesis requires further study.

In conclusion, our study showed that a considerable immune imbalance occurred in the follicular microenvironment of patients with AIT. Furthermore, to the best of our knowledge, this is the first demonstration of the activation of the intrafollicular IFNγ-CXCL9/10/11-CXCR3^+^ T lymphocyte cascade in patients with AIT.

## Data Availability Statement

The original contributions presented in the study are included in the article/supplementary material. Further inquiries can be directed to the corresponding authors.

## Ethics Statement

The studies involving human participants were reviewed and approved by Peking University Third Hospital Medical Science Research Ethics Committee. The patients/participants provided their written informed consent to participate in this study.

## Author Contributions

NH, HC, and JQ developed the conception of the study, and all authors contributed to the research discussion. NH, DL, and YL collected the samples. NH and HC performed the experiment and contributed to data analysis. NH wrote the initial draft of the paper, and all authors contributed to manuscript revision. All authors contributed to the article and approved the submitted version.

## Funding

This work was supported by the National Natural Science Foundation of China (Grant No. 81871212) and The Second Tibetan Plateau Scientific Expedition and Research (Grand No. 2019QZKK0607).

## Conflict of Interest

The authors declare that the research was conducted in the absence of any commercial or financial relationships that could be construed as a potential conflict of interest.

## Publisher’s Note

All claims expressed in this article are solely those of the authors and do not necessarily represent those of their affiliated organizations, or those of the publisher, the editors and the reviewers. Any product that may be evaluated in this article, or claim that may be made by its manufacturer, is not guaranteed or endorsed by the publisher.
